# Old nodes, new tricks: optimized methods for chronic wasting disease prion detection in preserved retropharyngeal lymph nodes

**DOI:** 10.1177/10406387261445902

**Published:** 2026-05-21

**Authors:** Avery Munster, Jennifer Høy-Petersen, Madison A. Davis, Sarah A. Tomke, Kevin D. Niedringhaus, Roderick B. Gagne, Michelle Gibison

**Affiliations:** University of Pennsylvania School of Veterinary Medicine, Philadelphia, PA, USA; Wildlife Futures Program, Department of Pathobiology, University of Pennsylvania School of Veterinary Medicine, New Bolton Center, Kennett Square, PA, USA; Wildlife Futures Program, Department of Pathobiology, University of Pennsylvania School of Veterinary Medicine, New Bolton Center, Kennett Square, PA, USA; Wildlife Futures Program, Department of Pathobiology, University of Pennsylvania School of Veterinary Medicine, New Bolton Center, Kennett Square, PA, USA; Wildlife Futures Program, Department of Pathobiology, University of Pennsylvania School of Veterinary Medicine, New Bolton Center, Kennett Square, PA, USA; Wildlife Futures Program, Department of Pathobiology, University of Pennsylvania School of Veterinary Medicine, New Bolton Center, Kennett Square, PA, USA; Wildlife Futures Program, Department of Pathobiology, University of Pennsylvania School of Veterinary Medicine, New Bolton Center, Kennett Square, PA, USA

**Keywords:** chronic wasting disease, real-time quaking induced conversion, white-tailed deer

## Abstract

Chronic wasting disease (CWD) is a highly transmissible, global prion disease of captive and wild cervids. The approved tests for the detection of the infectious CWD prion (PrP^CWD^) are ELISA and immunohistochemistry (IHC), but both have limitations and can yield discrepant results. ELISA requires a fresh or frozen medial retropharyngeal lymph node (RPLN); IHC requires the RPLN to be formalin-fixed and paraffin-embedded, thus requiring 2 different storage protocols. Real-time quaking-induced conversion (rtQuIC) is a sensitive amplification technique used commonly in research to detect prions. Here, we optimized a rtQuIC assay for CWD prion detection in formalin-fixed (FF) and formalin-fixed paraffin-embedded tissues (FFPE) from white-tailed deer in Pennsylvania with various prion protein (*PRNP*) genotypes. Our proposed protocol differs from other publications because it does not require hazardous solvents such as xylene. We found that FF RPLN samples can be tested by rtQuIC with up to 96% sensitivity and 100% specificity, and FFPE RPLN samples can be tested by rtQuIC with up to 98% sensitivity and 99% specificity for PrP^CWD^ detection. Our results are comparable to testing fresh or frozen tissue, and we did not find an effect of prion protein genotype on assay performance. This application of rtQuIC has the potential to improve surveillance and disease management in regions with limited access to cold storage and allow testing of RPLNs with discrepant PrP^CWD^ results when tissue of sufficient quality is unavailable.

Chronic wasting disease (**CWD**) is an established threat to cervids such as white-tailed deer (*Odocoileus virginianus*; hereafter **deer**), mule deer (*Odocoileus hemionus*), elk (*Cervus canadensis*), and moose (*Alces alces*) across North America that poses ecologic and potential public health risks.^41^ CWD is caused by a misfolded prion protein (**PrP^CWD^**) that originates from the normal cellular prion protein (**PrP^C^**) found in host cells.^36^ The accumulation of these pathogenic prions leads to neurodegeneration and eventual death in all infected animals.^37^ CWD has been identified in wild or captive cervids in 36 U.S. states,^35^ including Pennsylvania, where it has spread steadily since its detection in 2012.^16^ Disease Management Areas (**DMAs**) have expanded in response to new cases, but the rate of spread emphasizes the importance of accurate and timely detection. Although no human cases have been reported,^1^ the zoonotic potential remains uncertain,^40^ and the widespread consumption of deer elevates public health concerns. Effective surveillance and testing are critical tools for risk assessment and disease containment.

Although the current gold standard, or tests approved by the USDA, are immunohistochemistry (IHC) and ELISA, both have limitations in sensitivity and validated tissue types for testing (retropharyngeal lymph node (**RPLN**) and obex).^13^ Both tests require direct labeling of the infectious prion without amplification. IHC offers high specificity but requires formalin-fixed paraffin-embedded tissue (**FFPE**) and interpretation by a board-certified veterinary pathologist, whereas ELISA must be performed on fresh or frozen tissue and is primarily used as a screening tool in wild deer, with limited application for captive cervids.^27^ Real-time quaking-induced conversion (**rtQuIC**), a prion seeding amplification technique, has become a promising alternative test given its ability to detect minute amounts of PrP^CWD^ in various tissues, identifying CWD prions months before traditional methods.^3,19^ Despite the potential of the rtQuIC assay, its use has been largely limited to fresh or frozen tissues.^17^

Expanding the use of the rtQuIC assay to archival samples (such as formalin-fixed [**FF**] or FFPE tissues) enables retrospective prevalence estimates and the ability to map historical CWD spread. The utility of testing preserved tissues could also indirectly help improve overall tissue quality from free-ranging deer by increasing justification to preserve tissues in formalin as soon as possible and prevent further tissue degradation.^9^ Tissues from free-ranging deer and other wildlife are often more severely autolyzed (due to factors such as longer duration of time between mortality and tissue collection and increased exposure to harsh environmental conditions) compared with tissues from domestic animals that live in more controlled environments.^38^ Amplifying and detecting prion seeding in archival samples would address several key limitations of conventional methods such as IHC, which often lacks sensitivity in early or subclinical infections.^32^

Existing protocols for prion detection from FFPE tissues involve labor-intensive processing steps, including hazardous compounds such as xylene and graded alcohols, deparaffinization, and rehydration.^22,25^ Xylene is a volatile organic compound that is highly flammable, has significant risks associated with vapor inhalation, irritates skin and eyes, and requires special hazardous waste disposal.^24^ Using xylene and graded alcohol washes introduces the risk of toxicity, environmental hazards, and requires fume hoods. The ability to bypass these hazards and work outside a fume hood while still achieving prion amplification would offer methodologic and biosafety advantages.^24^

In addition to tissue type and processing, host genetic variation could affect the sensitivity of assays, as host genetic variation has been shown to influence the susceptibility to, and progression of, CWD.^2,4,12,26,29^ Studies have shown that non-synonymous genetic mutations that alter the amino acid sequence of the prion protein gene (***PRNP***) can slow prion conversion and extend the incubation period of disease in cervids.^2,6,12,18,26,29^ Comparatively, if *PRNP* gene mutations can slow prion conversion in the host, they could also slow or inhibit prion conversion in a prion amplification assay, such as the rtQuIC assay, and lead to false-negative results.^14^ Deer that are homozygous for the wildtype (wt) alleles (*95Q*/*96G*) have a shorter incubation period compared with individuals that are heterozygous for *95GH*, *96GS*, or *226QK*.^6,12^ One study—examining the detection of PrP^CWD^ in biopsy specimens of the rectoanal mucosa-associated lymphoid tissue by rtQuIC—reported negative correlations with the assay in samples that were either heterozygous at codon 96 (*96GS*) or homozygous (*96SS*),^14^ indicating that specific genotypes may result in discrepancies within the rtQuIC assay. Therefore, we evaluated *PRNP* genotypes alongside rtQuIC assay performance to better understand if genotypes impact PrP^CWD^ detection outcomes.

To date, no studies have reported the use of the rtQuIC assay on FF or FFPE tissues without including the additional processing steps mentioned previously. Here, we describe a novel method for detecting PrP^CWD^ seeding activity directly from FF and FFPE RPLNs without the removal of fixatives or paraffin. This simplified approach has the potential to streamline testing and expand testing capabilities for retrospective and large-scale surveillance.

## Materials and methods

### Sample collection and PrP^CWD^ detection

**Figure 1.**
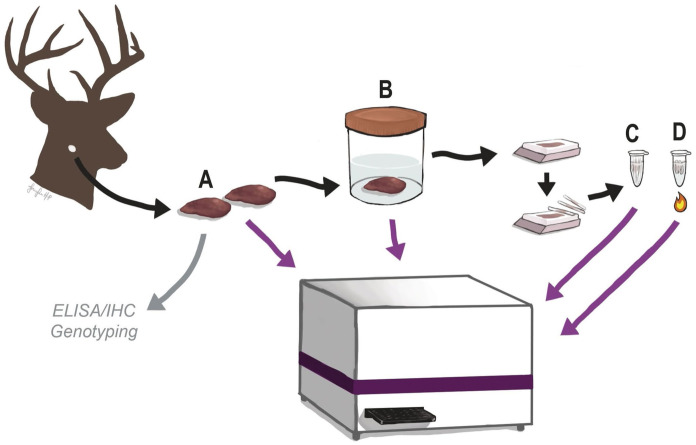
Schematic of the different tissue-preservation groups from white-tailed deer tested for PrP^CWD^. Retropharyngeal lymph nodes were tested as fresh-frozen (**A**), formalin-fixed (**B**), formalin-fixed paraffin-embedded (FFPE) scrolls (**C**), and heat-treated FFPE scrolls (**D**). The purple arrows point to a plate reader used to run real-time quaking-induced conversion assays to detect prions.

Paired RPLNs were collected from free-ranging deer in Pennsylvania, USA, between 2023 and 2024, either by certified CWD technicians from the Pennsylvania Game Commission or from the Penn Vet Wildlife Futures Program. Samples were opportunistically obtained postmortem from 58 hunter-harvested, 2 clinical CWD suspects, and 141 vehicle-struck deer. Given the high likelihood of tissue autolysis, obex is not routinely collected for surveillance in Pennsylvania. A total of 201 RPLNs were collected both within (*n* = 169) and outside (*n* = 32) of DMAs. Of these, 100 were PrP^CWD^-positive and 101 were PrP^CWD^-negative, confirmed by ELISA and/or IHC at the Pennsylvania Animal Diagnostic Laboratory (New Bolton Center, PA, USA), which is an American Association of Veterinary Laboratory Diagnostician–accredited diagnostic laboratory. The PrP^CWD^-positive samples had ELISA optical density (OD) values of 0.04 to >3.5 and a variety of IHC staining intensities. The OD value is relatively proportional to the quantity of target antibody in the sample, and a higher OD value in the TeSeE ELISA (Bio-Rad) indicates a higher concentration of prions. For each animal, one of the paired RPLNs was tested by ELISA and then stored at −80°C (fresh-frozen RPLN category); the contralateral RPLN was prepared for IHC (FF and FFPE categories). The contralateral RPLN was fixed in 10% neutral-buffered formalin for at least 24 h. The sample then was trimmed, processed routinely, and 5-μm sections were stained with a nonspecific prion antibody (Anti-Prion [99] research kit; Roche) and evaluated by a board-certified veterinary pathologist (KD Niedringhaus). To determine the suitability of testing preserved RPLNs for PrP^CWD^, we compared the initial ELISA and IHC results with the rtQuIC results for the same RPLN in the following formats: A) fresh-frozen, B) FF, C) FFPE without a heating step, and D) FFPE with a heating step (**Fig. 1**).

### Tissue preparation for fresh-frozen RPLNs

We weighed fresh-frozen RPLNs to 50 mg (± 5 mg) and transferred them to 2-mL reinforced lysing tubes with 2.8-mm ceramic beads (Omni International), which then were filled with 1× PBS to create a 10% weight/volume concentration and homogenized (Precellys Evolution homogenizer; Bertin Technologies) at 7500 rpm with 4 cycles of 30 s on, 30 s off, repeated twice.

### Tissue preparation from FF RPLNs

The time from formalin-fixation to testing by the rtQuIC assay for all RPLNs was 21–574 d at room temperature. We transferred each FF RPLN from the jar of formalin and weighed 50 mg (± 5 mg), which was transferred to a 2-mL reinforced lysing tube and homogenized with 1× PBS to create a 10% (wt/vol) homogenate, as described above.

### Tissue preparation from FFPE RPLN blocks

For the rtQuIC experiments, we removed four 5–10-µm thick FFPE tissue scrolls from the blocks using a microtome and placed them into a 2-mL microcentrifuge tube. A new blade was placed on the microtome between each tissue block to prevent contamination between blocks, and the first scroll from each block was always discarded to avoid any possible surface contamination.^22^

We homogenized the 4 tissue scrolls, as described above, either directly or after applying a heat treatment to melt the paraffin wax. The heat treatment was included to determine if rtQuIC assay performance differed when we increased tissue concentration by melting a portion of the paraffin wax coating. For the heat treatment protocol, we added 1 mL of 1× PBS to the 2-mL microcentrifuge tube that contained the tissue scrolls and placed it on a 100°C heating block (Digital heating shaking drybath; ThermoFisher) and agitated it at 650 rpm for 5 min. After 5 min, the tube was vortexed to ensure equal distribution of 1× PBS around the tissue and returned to the heating block for another 5 min. After a total of 10 min, the tissue was transferred to a new microcentrifuge with 1 mL of 1× PBS (using a new pair of disposable forceps for each sample to avoid cross-contamination). The 10-min heating and vortex procedure was repeated. Finally, the tubes were cooled to room temperature, and the remaining scroll pieces were trimmed to 50 mg (± 5 mg) and homogenized, as described previously.

### Serial dilutions of RPLN homogenates

Before performing the rtQuIC assay, we diluted the RPLN homogenate in each preservation category using a commercial CWD sample dilution buffer (0.1% sodium dodecyl sulfate, 10× PBS; VMRD]). We refer to 5 µL of the 10% weight/volume homogenate combined with 45 µL of buffer as a 10^–1^ dilution in this paper.

The fresh-frozen RPLNs were tested at a dilution of 10^–3^ in replicates of 3, which our previous experiments and other publications have found to be ideal.^11,23^ For the other RPLN preservation categories, we conducted optimization experiments with RPLNs from the same individuals (2 PrP^CWD^-positive, and 2 PrP^CWD^-negative) in replicates of 6. We optimized a dilution that would yield a consistently high amyloid formation rate (**AFR**) for the positive samples, and a consistently low or no AFR for a negative sample. Given our suspicion that formalin may inhibit the rtQuIC assay and require a higher dilution factor, we intentionally conducted the first experiment with the FF RPLNs and covered a wider range of dilutions (10^–1^–10^–8^). Because the lower dilutions appeared to work well for this category, we only included dilutions 10^–1^–10^–4^ for the FFPE preservation categories. A Kruskal–Wallis chi-squared test and pairwise comparisons with a Bonferroni correction were performed on the serial dilutions for the 3 preservation categories to validate the chosen dilutions for our study.

### rtQuIC assay conditions

We conducted the rtQuIC assay reaction using commercial substrate (CWD amplification reagent, Syrian hamster substrate; VMRD) and reaction buffer (5× PBS, pH 7.4, 0.85 M NaCl, 5 mM EDTA, and 50 µM thioflavin T; VMRD), which was added to a 96-well plate (Item 655096; Greiner Bio-One) at a volume of 98 µL with 2 µL of sample for a total volume of 100 µL per well.^19^

The rtQuIC assay was run (FLUOstar plate reader; BMG Labtech), as described previously,^23^ with a temperature of 42°C for 250 cycles, or a duration of 62.5 h set to 700 rpm with 60-s shake/60-s rest cycles, 4-mm double-orbital fluorescent scans every 15 min at an excitation of 450 nm, and emission of 480 nm. We adjusted the plate reader gain setting based on the highest fluorescing positive control (RPLN from a known PrP^CWD^-positive free-ranging deer in Pennsylvania) on the plate, with values of 1,300–1,700. The positive and negative controls on each plate were fresh-frozen RPLNs from known PrP^CWD^-positive and -negative deer in Pennsylvania and well-characterized previously by rtQuIC testing.

### rtQuIC data analysis

To determine whether a sample was positive or negative by rtQuIC, we calculated a fluorescence threshold, as described previously.^8,15,23,43^ In summary, baseline fluorescence was calculated as the mean of the fluorescence of each well in the first 3 cycle reads that occur after the initial quaking (cycles 2–4 when running a protocol in the BMG Labtech microplate reader control software) and the threshold was calculated by adding 10 SDs. The time-to-threshold in hours and the determined AFR, or 1/time-to-threshold, was determined for all wells on the 96-well plate. Time-to-threshold calculations were done using Excel (v.2603; Microsoft) with input into Omega Data Analysis software (BMG Labtech).

The number of replicates that crossed a calculated threshold by 192 cycles (48 h) was used to determine the PrP^CWD^ status of a sample. Fresh-frozen and control samples were considered positive when the fluorescence of at least 2 of the 3 wells crossed the threshold within the 0.02 AFR cutoff value (48 h). All FF and FFPE samples were tested in replicates of 6 and were considered positive if 3 replicates crossed the defined threshold of 0.02 AFR (48 h). Conditions can be adjusted by the laboratory or governing wildlife management agency to optimize rtQuIC assay performance based on desired outcomes, whether the goal is to maximize sensitivity to identify all potential positive animals or to maximize specificity to reduce the likelihood of false-positives.

### DNA extraction and sequencing

Genomic DNA was extracted from fresh-frozen deer RPLNs (DNeasy blood & tissue kit; Qiagen), following the manufacturer’s recommended protocol. A 938-bp region of the *PRNP* gene was amplified by PCR, using a published protocol and primer set (WTDPRNP-F: 5′-TGTTTATAGCTGATGCCACTGC-3′; WTDPRNP-R: 3′-ACACCACCACTACAGGGC-5′).^12^ Amplified products were run on a 1% agarose gel (Invitrogen, ThermoFisher) to confirm the presence of the amplicon product before sequencing.^6,12^ Sample purification and Sanger sequencing of PCR amplicons (Eurofins Genomics, Louisville, KY, USA) were performed using both the forward and reverse primers.

The software Geneious Prime v.2025.1.2 (GraphPad) was used to edit AB1 files from both the forward and reverse reads of each sample. Each sequence was trimmed and aligned to a 771-bp reference *PRNP* sequence (GenBank AF156185.1).^34^ IUPAC ambiguous nucleotide (nt) codes were used when a sample was heterozygous for 2 nts at a given position. Sequences were then exported in FASTA file format for haplotyping.

Haplotypes were generated in the software DnaSP (v.6.12.03; Universitat de Barcelona) by running 100,000 Markov chain Monte Carlo iterations with a burn-in of 10,000 on unphased sequences. Haplotypes generated from DnaSP6 were compared and identified to *PRNP* haplotypes described in the literature.^6,7,10,30,31,33^ Both haplotype and proteoform frequencies were calculated for the sample set, allowing for the identification of important deer *PRNP* polymorphisms (*96QH*, *96GS*, *226QK*).

### Fisher exact test for genotype-associated discrepancies

Any discrepancies between the ELISA and IHC results and the rtQuIC assay results were further explored to determine if genotype variations impacted our assay performance. Given variations within the *PRNP* gene at amino acids *95QH, 96GS*, and *226QK*, a Fisher exact test was used to investigate whether an association between discrepant rtQuIC results could be explained by *PRNP* polymorphisms. For each tissue treatment group, a separate Fisher exact test was used to compare the frequency of agreeing and discrepant rtQuIC results between wildtype (proteoforms A) and non-wildtype samples. Wildtype samples were classified as animals that expressed only proteoform A across both *PRNP* genes, whereas non-wildtype animals expressed any of the following proteoform combinations (A/C, A/F, A/K, C/C, or C/K).

## Results

### Serial dilutions of RPLN homogenates

**Figure 2.**
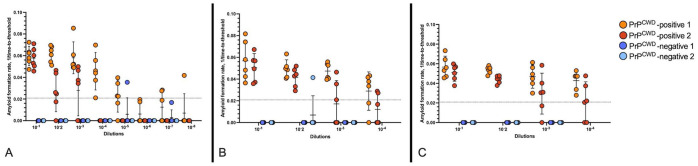
Amyloid formation rates of preserved retropharyngeal lymph nodes (RPLNs) from white-tailed deer tested by real-time quaking-induced conversion assay for PrP^CWD^. The graphs show amyloid formation rates for 2 PrP^CWD^-positive and 2 PrP^CWD^-negative RPLNs at different dilutions. **A.** Formalin-fixed RPLN. **B.** Non–heat-treated, formalin-fixed paraffin-embedded (FFPE) RPLN. **C.** Heat-treated FFPE RPLN. Dotted horizontal line = the amyloid formation rate threshold used to differentiate between positive and negative PrP^CWD^ results.

We observed significant differences in AFR among dilutions for FF RPLNs (Kruskal–Wallis: *X*^2^ = 56.5, df = 7, *p* < 0.001; **Fig. 2A**), indicating that some dilutions were suboptimal. Pairwise comparisons among dilutions 10^–1^–10^–3^ resulted in no significant findings (*p* = 0.63–1.00; **Suppl. Table 1**). In contrast, significant differences were observed among dilutions 10^–4^–10^–8^ compared with a 10^–1^ dilution (*p* = 0.000–0.035), indicating inhibited or slower amplification success in less-concentrated samples. Therefore, we elected to move forward with 10^–1^ dilutions for the FF category.

Similar trends were observed for both non–heat-treated (**Fig. 2B**) and heat-treated FFPE (**Fig. 2C**) RPLNs as with the FF RPLNs. We observed significant differences in AFR among all dilutions for non–heat-treated FFPE RPLNs (Kruskal–Wallis: *X*^2^ = 18.3, df = 3, *p* < 0.001) and heat-treated FFPE RPLNs (Kruskal–Wallis: *X*^2^ = 12.8, df = 3, *p* = 0.005). We did not find significant differences in AFR for pairwise comparisons among dilutions 10^–1^–10^–3^ (*p* = 1.00) for the non–heat-treatment group, but a significant difference was observed in the 10^–4^ dilution compared with the 10^–2^ dilution (*p* = 0.004). For the heat-treated group, we did not find significant differences in AFR for pairwise comparisons among dilutions 10^–1^–10^–4^ compared with the 10^–2^ dilution (*p* = 0.052–0.960; Suppl. Table 1). Based on these results, we selected a 10^–2^ dilution for all subsequent analyses for both FFPE groups, given a narrower range of AFRs within a shorter assay run time.

### rtQuIC assay results

From the complete dataset for each tissue type, we compared several combinations of positive replicates determined by AFR and their resulting sensitivity and specificity (**Table 1**). For our laboratory use and all future analyses in our study, we selected a 0.02 AFR (48 h) cutoff requiring positivity in 3 of 6 wells, given the high specificity across tissue types and our priority of minimizing potential false-positive results (shaded column in Table 1, see **Supplemental Excel rtQuIC data for all results**). We selected this cutoff for consistency and to maximize specificity across all treatment groups. Of the confirmed PrP^CWD^-positive fresh-frozen RPLN samples, 99 of 100 tested positive (99% sensitivity)—using the criteria of 2 of 3 replicates crossing the AFR cutoff of 0.02 (48 h)—and all 101 fresh-frozen confirmed PrP^CWD^-negative RPLNs tested negative (100% specificity) by rtQuIC.

**Table 1.** Comparison of real-time quaking-induced conversion (rtQuIC) assay results for formalin-fixed (FF), heat-treated formalin-fixed paraffin-embedded (FFPE), and non–heat-treated samples with different well and time-to-threshold cutoffs for retropharyngeal lymph nodes from wild white-tailed deer in Pennsylvania, USA. We selected the cutoff criteria in the shaded column.

Sensitivity and specificity	rtQuIC PrP^CWD^-positive result criteria			
	2 of 6 wells 0.02 AFR (48 h)	2 of 6 wells 0.016 AFR (62.5 h)	3 of 6 wells 0.02 AFR (48 h)	3 of 6 wells 0.016 AFR (62.5 h)
FF sensitivity	95/100	96/100	92/100	94/100
FF specificity	100/101	96/101	101/101	101/101
FFPE (no heat) sensitivity	94/100	94/100	92/100	94/100
FFPE (no heat) specificity	99/101	96/101	100/101	98/101
FFPE (heat) sensitivity	97/100	98/100	96/100	97/100
FFPE (heat) specificity	96/101	95/101	100/101	99/101

AFR = amyloid formation rate; PrP^CWD^ = infectious chronic wasting disease prion protein.

For the FF samples, 92 of 100 of the confirmed PrP^CWD^-positive RPLNs tested positive (92% sensitivity); 8 samples remained below the detection threshold and were considered false-negatives. Because the RPLNs in this group were stored in formalin for 21–574 d before the rtQuIC assay was conducted, we compared the formalin storage duration between the false-negatives and the true-positives. We found no statistically significant difference (Kruskal–Wallis: *X*^2^ = 0.09, df = 1, *p* = 0.760) in time spent in formalin between RPLNs that tested true-positive (*n* = 92, range = 23–574 d, median = 196, interquartile range = 169–258) and false-negative (*n* = 8, range = 50–518 d, median = 213, interquartile range = 160–290). All 101 of the confirmed PrP^CWD^-negative FF RPLNs tested negative (100% specificity).

Without heat treatment, 92 of 100 confirmed PrP^CWD^-positive FFPE RPLNs tested positive (92% sensitivity); 8 did not reach the established threshold, and 4 of these were the same RPLNs that tested negative by rtQuIC in the heat-treatment FFPE category (samples 32, 39, 121, 124). Of the 101 confirmed PrP^CWD^-negative samples, 100 non–heat-treated FFPE RPLNs tested negative, with 1 false-positive sample (99% specificity). The only difference between the non-heated and heated FFPE categories with the criteria we selected was that the heat-treated FFPE RPLNs had a higher sensitivity of 96 of 100 (96%). The specificity was the same for both groups (100 of 101, 99%), with the same sample ID (sample 193) being false-positive.

### Sequencing and genetic analysis

We successfully sequenced the *PRNP* gene from 192 of 201 deer individuals from their respective fresh-frozen RPLNs. Of these individuals, 98 tested positive for PrP^CWD^; 94 did not have PrP^CWD^ detected by ELISA and/or IHC. Nine individuals did not have viable DNA to produce a sequenced product, as degradation of the genomic DNA was observed. From the 192 samples, we identified 16 previously described haplotypes with 5 distinct proteoforms expressed and their respective frequencies (**Suppl. Fig. 1**, **Suppl. Tables 2, 3**). Haplotype A, which reflects wildtype *PRNP*, had the highest frequency (46.4%) among the haplotypes identified. Other notable haplotypes, including C, F, K, and M, had frequencies of 8.8%, 0.8%, 2.3%, and 1.3%, respectively. Although haplotype A was the most common haplotype identified in our sample set, proteoform A was also the most common among the 5 proteoforms identified, with a frequency of 81.5%. Proteoforms C, F, K, and M were expressed in much lower frequencies, at 14.1%, 0.8%, 2.3%, and 1.3%, respectively (Suppl. Fig. 1).

Among our 16 identified haplotypes, we identified 3 important and previously described *PRNP* genotypes in our sample set that are known to affect disease outcome in deer and discrepancies among tests. These genotypes include variations at amino acid positions *95H* (nt position 285), *96S* (nt position 286), and *226K* (nt position 676; **Table 2**). These amino acid variations correspond to haplotypes and proteoforms C, F, and K, respectively. At *95H* and *226K*, we observed 3 PrP^CWD^-positive individuals that were heterozygous *95QH* and 9 PrP^CWD^-negative individuals that were heterozygous *226QK*. No individuals were observed to be homozygous for either *95H* or *226K*. However, at codon 96, we identified 9 PrP^CWD^-positive and 32 PrP^CWD^-negative individuals to be heterozygous *96GS*. We also observed 5 PrP^CWD^-negative individuals to be homozygous for *96S*.

**Table 2.** Total observed prion protein genotypes. Four important non-synonymous, single-nucleotide polymorphisms in the prion protein (*PRNP*) gene were observed in white-tailed deer from Pennsylvania, USA. The corresponding wildtype (wt) amino acid at each codon is documented. Each codon accounts for the total number of samples that were sequenced (*n* = 192).

Codon	Amino acid change	*PRNP* genotype	PrP^CWD^-positive	PrP^CWD^-negative	Total
95	Glutamine → Histidine	95QQ (wt)	95	94	189
		95QH	3	0	3
96	Glycine → Serine	96GG (wt)	89	57	146
		96GS	9	32	41
		96SS	0	5	5
226	Glutamine → Lysine	226QQ (wt)	98	85	183
		226QK	0	9	9

PrP^CWD^ = infectious chronic wasting disease prion protein.

### Linking genotype to discrepant rtQuIC results

Across all tissue treatment groups, 12 samples resulted in PrP^CWD^ status disagreements between the rtQuIC results compared with the ELISA or IHC results (**Table 3**). We found 6 discrepant samples that were associated with wildtype *PRNP*, or proteoform A. The remaining 6 discrepant samples were associated with non-wildtype genotypes. These samples correspond with variations in the *PRNP* gene at codon *95QH* (*n* = 1) and *96GS* (*n* = 5), reflecting proteoforms F and C, respectively.

**Table 3.** Twelve samples (shaded) resulted in discrepancies in the real-time quaking-induced conversion (rtQuIC) assay compared to the ELISA or immunohistochemistry (IHC) result, either in the fresh-frozen retropharyngeal lymph node (RPLN), formalin-fixed (FF) RPLN, or heat-treated and non–heat-treated formalin-fixed paraffin-embedded (FFPE) RPLNs from white-tailed deer.

ID	Genotype	PrP^CWD^	ELISA OD	Discrepancies in rtQuIC result			
				Fresh-frozen	FF	FFPE (no heat)	FFPE (heat)
10	Wt	Positive	1.70	TP	TP	FN	TP
32	96GS	Positive	1.85	TP	FN	FN	FN
35	95QH	Positive	0.97	TP	FN	FN	TP
39	Wt	Positive	0.07	TP	FN	FN	FN
106	Wt	Positive	3.163	TP	FN	FN	TP
110	96GS	Positive	0.21	FN	FN	TP	TP
113	Wt	Positive	0.10	TP	FN	TP	TP
115	96GS	Positive	0.13	TP	FN	TP	TP
121	Wt	Positive	0.70	TP	TP	FN	FN
124	Wt	Positive	0.07	TP	FN	FN	FN
129	96GS	Positive	0.28	TP	TP	FN	TP
193	96GS	ND	0.02	TN	TN	FP	FP

FN = false-negative; FP = false-positive; ND = not detected by ELISA or IHC; PrP^CWD^ = infectious chronic wasting disease prion protein; TN = true-negative; TP = true-positive; Wt = wildtype.

### Fisher exact test for discrepancies

Genotype was not associated with rtQuIC assay discrepancies (*p* = 0.070–0.468, using a Bonferroni corrected alpha because of multiple comparisons; **Suppl. Table 4**) in any group except for the CWD-positive FF RPLNs (*p* = 0.010). However, this significant finding may reflect the unequal sample size between wildtype and non-wildtype deer included in our comparison and not an influence from the genotypes themselves, given that the number of false-positive discrepancies between the 2 groups was the same (*n* = 4). Overall, we did not find substantial evidence that clearly indicates that *PRNP* genotypes were associated with reduced rtQuIC assay sensitivity or specificity in our study.

## Discussion

We found that PrP^CWD^ remains detectable in FF and FFPE tissue even in the absence of conventional protocols that require xylene and/or graded alcohol washes. Prion amyloid seeding activity appears to persist despite fixation and paraffin embedding and broadens opportunities for detection and analysis of preserved tissue samples without the need for more expensive and potentially hazardous tissue processing.

In our laboratory, a 0.02 AFR (48 h) cutoff with 3 of 6 well positivity criteria provided the highest specificity while still maintaining sensitivity. However, other laboratories or management agencies may wish to optimize rtQuIC assay conditions differently depending on sample availability and whether minimizing false-negatives or false-positives is a higher priority. Parameters such as AFR cutoff, number of replicate wells, and pretreatment protocols could be adjusted to meet specific agency needs. With our AFR cutoff and number of wells, we observed that FFPE RPLNs undergoing heat treatments to mechanically facilitate wax removal had increased sensitivity (96%) while maintaining the same specificity (99%) as non–heat-treated FFPE RPLNs. Notably, non–heat-treated FFPE RPLNs still yielded a high sensitivity (92%) and equal specificity (99%), which presents significant practical advantages and reduced cross-contamination risk because of eliminating the handling of hot wax and tissue when transferring samples between tubes. These results indicate a high level of PrP^CWD^ detection accuracy for rtQuIC applied to FF and FFPE RPLN tissue.

We theorize 2 potential causes of false-negatives: 1) those related to sample preservation and 2) those related to low and/or unequally distributed PrP^CWD^ concentration. Sample preservation, such as formalin fixation and/or embedding in paraffin wax, may be a contributing factor, given that the highest number of false-negatives were FF and FFPE non–heat-treated RPLNs (*n* = 8 in both, but some from different individuals). Despite the difficulty associated with inactivating or removing infectious prions, it is possible that extended fixation in formaldehyde forms covalent bonds or “cross-linking” to amino acids that inhibits binding between the rtQuIC assay substrate and prions in the tissue.^21,28,39,42^ Even though we did not find a statistically significant difference in time spent in formalin between RPLNs that tested true-positive and false-negative in our study, cross-linking could have impacted assay sensitivity. We still maintain that a sensitivity of 92% for FF RPLNs may be adequate in certain scenarios, especially where other tissue may be unavailable. Continued trials should be conducted to evaluate the ability of the rtQuIC assay to detect PrP^CWD^ in RPLNs that have been formalin-fixed for ≥1.5 y to determine if duration in formalin is a critical limitation or not.

Considering the difference in sensitivity between the fresh-frozen RPLN and the contralateral FF and FFPE RPLN, another explanation for false-negatives could be because of unequally distributed PrP^CWD^ concentration in the 2 RPLNs.^5^ RPLN that was fixed in formalin and later embedded in paraffin wax lacked or contained a lower concentration of infectious prions and thus may have yielded a false-negative rtQuIC result when the contralateral fresh-frozen RPLN tested true-positive. Similarly, one of the fresh-frozen RPLNs (sample 110) yielded a false-negative result by the rtQuIC assay despite being positive by ELISA and IHC. A low concentration of the target prion protein is a potential cause, as is also indicated by the low OD value in the ELISA (sample 110 had an OD of 0.21).

In the heat-treated FFPE RPLN group, the number of false-negatives was lower (*n* = 4) than in the non–heat-treated group (*n* = 8). This could suggest that the tissue scrolls included in the heat-treatment experiment were taken from an area of the paraffin block that overall contained more RPLN tissue, or that the sections of RPLN included had a higher concentration of infectious prions than those used in our non–heat-treatment experiment. However, another explanation is that the heat treatment denatured or removed substances in the formalin or paraffin wax that inhibit the rtQuIC assay. Heat can often inactivate enzymes or other molecules that interfere with assays, improving overall sensitivity.^9^ To summarize, the rtQuIC assay may not have amplified for FF or FFPE RPLNs because of inhibitors that could have interacted with the assay, or possibly because of a low concentration in the selected section of the tissue or unequal distribution of prions across RPLNs.^5^ This biological variability can lead to false-negatives even when a highly sensitive assay is used.

Only one false-positive RPLN was found across the different tissue-processing categories (sample 193 for the heat-treated and non–heat-treated group). One explanation could be that this individual was in the early stage of the disease with little to no accumulation of infectious prions in the RPLNs. If this was the case, the preserved RPLN that tested positive may have contained a higher prion concentration than the contralateral RPLN, resulting in seeding and a positive rtQuIC test result. Again, this could indicate that the contralateral fresh-frozen RPLN (that also tested negative by ELISA with an OD of 0.02) may not have contained infectious prions, or that the concentration was too low to achieve seeding amplification by rtQuIC. This would mean that the heat-treated and non–heat-treated FFPE false-positives reflected true early PrP^CWD^ detection, although notably this sample was negative on the FF tissue of the same RPLN. Another explanation could be cross-contamination associated with the microtome or mechanically removing paraffin.^20^ The use of thermomixers and the additional sample handling introduce the increased possibility of material splashing between tubes, which presents not only a risk of contamination but could also pose a biosafety hazard, emphasizing the need to conduct the protocol in a space where surfaces can easily be decontaminated. However, both heat treatment and non-heat treatment of the FFPE RPLN yielded a false-positive result. Therefore, another potential source of contamination is the use of a microtome to section scrolls of tissue from the paraffin block, which could help explain the false-positives observed in both FFPE categories. Although the blades were changed between paraffin-embedded RPLN blocks and the machine was wiped down to mechanically remove residue, the blocks were handled on the same surface. Given the demonstrated hardiness of prions, it is important to establish a protocol that does not introduce a higher likelihood of cross-contamination between samples or serve as a biosafety risk to the laboratory personnel or diagnostician. Our protocol minimizes sample contamination risk and accounts for diagnostician safety.

To determine if discrepant results were a consequence of genotype inhibition by altering amyloid formation in the rtQuIC assay, we analyzed the *PRNP* gene sequence. The *PRNP* gene plays an important role in novel tissue optimization for both routine and research CWD assays, given that certain species and their associated variations in the *PRNP* gene can cause discrepant results.^14^ We identified several common genotypes that play a role in altered disease incubation period and/or effective PrP^CWD^ detection. We observed 12 discrepant results among 3 distinct genotypes, including wildtype (*n* = 6), *Q95H* (*n* = 1), and *G96S* (*n* = 5). Our results suggest that different genotypes do not affect the efficacy of FF or FFPE RPLNs in the rtQuIC assay, given that none of the wildtype genotypes was considered more likely to yield discrepant results (false-positive or false-negative).

Our findings indicate that prion amyloid seeding remains detectable by the rtQuIC assay of FF and FFPE tissue, with high sensitivity and specificity, and without protocols for reversal of cross-linking. Our results offer a foundation for more efficient and safer methodologies for PrP^CWD^ detection in preserved tissue, with implications for both confirmatory and proactive PrP^CWD^ detection.

## Supplemental Material

sj-pdf-1-vdi-10.1177_10406387261445902 – Supplemental material for Old nodes, new tricks: optimized methods for chronic wasting disease prion detection in preserved retropharyngeal lymph nodes Supplemental material, sj-pdf-1-vdi-10.1177_10406387261445902 for Old nodes, new tricks: optimized methods for chronic wasting disease prion detection in preserved retropharyngeal lymph nodes by Avery Munster, Jennifer Høy-Petersen, Madison A. Davis, Sarah A. Tomke, Kevin D. Niedringhaus, Roderick B. Gagne and Michelle Gibison in Journal of Veterinary Diagnostic Investigation

sj-xlsx-2-vdi-10.1177_10406387261445902 – Supplemental material for Old nodes, new tricks: optimized methods for chronic wasting disease prion detection in preserved retropharyngeal lymph nodes Supplemental material, sj-xlsx-2-vdi-10.1177_10406387261445902 for Old nodes, new tricks: optimized methods for chronic wasting disease prion detection in preserved retropharyngeal lymph nodes by Avery Munster, Jennifer Høy-Petersen, Madison A. Davis, Sarah A. Tomke, Kevin D. Niedringhaus, Roderick B. Gagne and Michelle Gibison in Journal of Veterinary Diagnostic Investigation
