# Supplementary material for: Old nodes, new tricks: optimized methods for chronic wasting disease prion detection in preserved retropharyngeal lymph nodes
Source: J Vet Diagn Invest. 2026 May 21:10406387261445902. Online ahead of print. doi: 10.1177/10406387261445902 (PMC13194330; doi:10.1177/10406387261445902)
Supplement: sj-pdf-1-vdi-10.1177_10406387261445902 – Supplemental material for Old nodes, new tricks: optimized methods for chronic wasting disease prion detection in preserved retropharyngeal lymph nodes [file sj-pdf-1-vdi-10.1177_10406387261445902.pdf]

JVDI: Supplemental material

Munster A, et al. Old nodes, new tricks: optimized methods for chronic wasting disease prion detection in preserved retropharyngeal lymph nodes

Supplemental Table 1. Results from Kruskal–Wallis tests and post hoc pairwise comparisons testing significant differences among amyloid formation rates at various sample dilutions. Tests were performed for formalin-fixed retropharyngeal lymph nodes (RPLNs), non–heat-treated, formalin-fixed paraffin-embedded RPLNs, and heat-treated, formalin-fixed paraffin-embedded RPLNs.

Formalin-fixed RPLNs			
Kruskal–Wallis	Test statistic	df	*p* value
	56.47	7	<0.001

Wilcoxon rank sum pairwise comparisons of dilutions 10^{-1}–10^{-7}							
	10^{-1}	10^{-2}	10^{-3}	10^{-4}	10^{-5}	10^{-6}	10^{-7}
10^{-1}	1.000	—	—	—	—	—	—
10^{-2}	0.631	1.000	—	—	—	—	—
10^{-3}	0.035	1.000	1.000	—	—	—	—
10^{-4}	0.001	0.034	0.052	1.000	—	—	—
10^{-5}	0.006	0.003	0.011	1.000	1.000	—	—
10^{-6}	0.001	0.018	0.017	1.000	1.000	1.000	—
10^{-7}	0.000	0.004	0.011	0.794	1.000	1.000	1.000

Non–heat-treated, formalin-fixed paraffin-embedded RPLNs			
Kruskal–Wallis	Test statistic	df	*p* value
	18.33	3	<0.001
**Wilcoxon rank sum pairwise comparisons of dilutions 10^{-1}–10^{-3}**			
	10^{-1}	10^{-2}	10^{-3}
10^{-1}	1.000	—	—
10^{-2}	0.116	1.000	—
10^{-3}	0.002	0.004	0.728

Heat-treated, formalin-fixed paraffin-embedded RPLNs			
Kruskal–Wallis	Test statistic	df	*p* value
	12.77	3	0.005
**Wilcoxon rank sum pairwise comparisons of dilutions 10^{-1}–10^{-7}**			
	10^{-1}	10^{-2}	10^{-3}
10^{-1}	1.000	—	—
10^{-2}	0.145	0.960	—
10^{-3}	0.021	0.052	1.000

Dash (—) = excluded to avoid repeating the same values across multiple fields.

Supplemental Table 2. Nucleotide positions and changes that occur for each haplotype compared to haplotype A (wildtype).

Nucleotides														*N*	Frequency, %
Amino acid position	20	51	81	95	96	100	108	126	139	146	156	185	226		
Nucleotide position	60	153	243	285	286	299	324	378	417	438	468	555	676		
Haplotype															
A	C	C	T	A	G	G	A	G	A	C	C	C	C	178	46.4
B	C	C	T	A	G	G	A	G	A	C	C	T	C	53	13.8
C	C	C	T	A	A	G	A	G	A	C	C	T	C	34	8.9
D	C	T	T	A	G	G	A	G	A	C	C	C	C	46	11.9
E	C	C	T	A	G	G	A	G	A	T	C	C	C	17	4.4
F	T	C	T	C	G	G	A	G	A	C	C	C	C	3	0.8
I	C	C	A	A	A	G	A	G	A	C	C	T	C	6	1.6
K	T	C	T	A	G	G	A	G	A	C	C	C	A	9	2.3
M	C	C	T	A	G	A	A	G	A	C	C	C	C	5	1.3
P	C	C	T	A	A	G	A	G	A	C	C	C	C	10	2.6
T	C	T	T	A	G	G	A	A	A	C	C	C	C	3	0.8
PRNP-Odvi27	C	C	A	A	A	G	A	G	A	C	C	C	C	4	1.0
PRNP-Odvi32	C	T	T	A	G	G	A	G	A	T	C	C	C	1	0.3
J	C	C	T	A	G	G	G	G	A	C	C	C	C	5	1.3
G	T	C	T	A	G	G	A	G	A	C	C	C	C	8	2.1
Heaton	C	C	T	A	G	G	A	G	G	C	T	C	C	2	0.5

Nucleotides highlighted in yellow indicate a single-nucleotide polymorphism (SNP) compared to the wildtype nucleotide and associated position. *N* = the number of alleles associated with each of the 16 haplotypes identified. The frequency of each haplotype was calculated by dividing the number of observed haplotypes by the total number of alleles in the sample set.

Supplemental Table 3. Amino acid changes and their respective codons that occurred for each haplotype documented when compared to haplotype A (wildtype).

Amino acids													PF
Amino acid position	20	51	81	95	96	100	108	126	139	146	156	185	226	
NT position	60	153	243	285	286	299	324	378	417	438	468	555	676	
Haplotype														
A	D (Asp)	R (Arg)	G (Gly)	Q (Gln)	G (Gly)	S (Ser)	P (Pro)	G (Gly)	R (Arg)	N (Asn)	N (Asn)	I (Ile)	C (Cys)	A
B	D	R	G	Q	G	S	P	G	R	N	N	I	C	A
C	D	R	G	Q	S (Ser)	S	P	G	R	N	N	I	C	C
D	D	R	G	Q	G	S	P	G	R	N	N	I	C	A
E	D	R	G	Q	G	S	P	G	R	N	N	I	C	A
F	D	R	G	H (His)	G	S	P	G	R	N	N	I	C	F
I	D	R	G	Q	S (Ser)	S	P	G	R	N	N	I	C	C
K	D	R	G	Q	G	S	P	G	R	N	N	I	A (Ala)	K
M	D	R	G	Q	G	N (Asn)	P	G	R	N	N	I	C	M
P	D	R	G	Q	S (Ser)	S	P	G	R	N	N	I	C	C
T	D	R	G	Q	G	S	P	G	R	N	N	I	C	A
PRNP-Odvi27	D	R	G	Q	S (Ser)	S	P	G	R	N	N	I	C	C
PRNP-Odvi32	D	R	G	Q	G	S	P	G	R	N	N	I	C	A
J	D	R	G	Q	G	S	P	G	R	N	N	I	C	A
G	D	R	G	Q	G	S	P	G	R	N	N	I	C	A
Heaton	D	R	G	Q	G	S	P	G	R	N	N	I	C	A

NT = nucleotide; PF = proteoform. Amino acid codes highlighted in yellow indicate a single-nucleotide polymorphism (SNP) compared to wildtype. Most SNPs documented are reflective of proteoform A, as a synonymous mutation occurred and therefore do not change the protein being expressed. Proteoforms C, F, K, and M all report at least one non-synonymous mutation, leading to a change in the amino acid at a specific codon and thus altering the protein being expressed.

Supplemental Table 4. Fisher exact tests to determine if there was an association between chronic wasting disease prion protein (PrP^{CWD}) genotype and discrepant real-time quaking-induced conversion assay results with initial ELISA and/or immunohistochemistry results in white-tailed deer.

Discrepant PrP^{CWD}-positive	FN (wt)	FN (non-wt)	TP (wt)	TP (non-wt)	*p* value
Fresh/frozen	0	1	85	12	0.133
FF	4	4	81	9	0.010*
FFPE non-heat	5	3	80	10	0.440
FFPE heat	3	1	82	12	0.070
Discrepant PrP^{CWD}-negative	FP (wt)	FP (non-wt)	TN (wt)	TN (non-wt)	*p* value
FFPE non-heat	0	1	50	43	0.468
FFPE heat	0	1	50	43	0.468

FF = formalin-fixed; FFPE = formalin-fixed paraffin-embedded; FN = false-negative; FP = false-positive; TN = true-negative; TP = true-positive. Genotypes are referred to as non-wildtype (non-wt) or wildtype (wt). The far-left column indicates whether the discrepant result was found in retropharyngeal lymph nodes from PrP^{CWD}-positive or PrP^{CWD}-negative deer, and within which tissue-preservation group.

* Statistically significant with a Bonferroni correction.

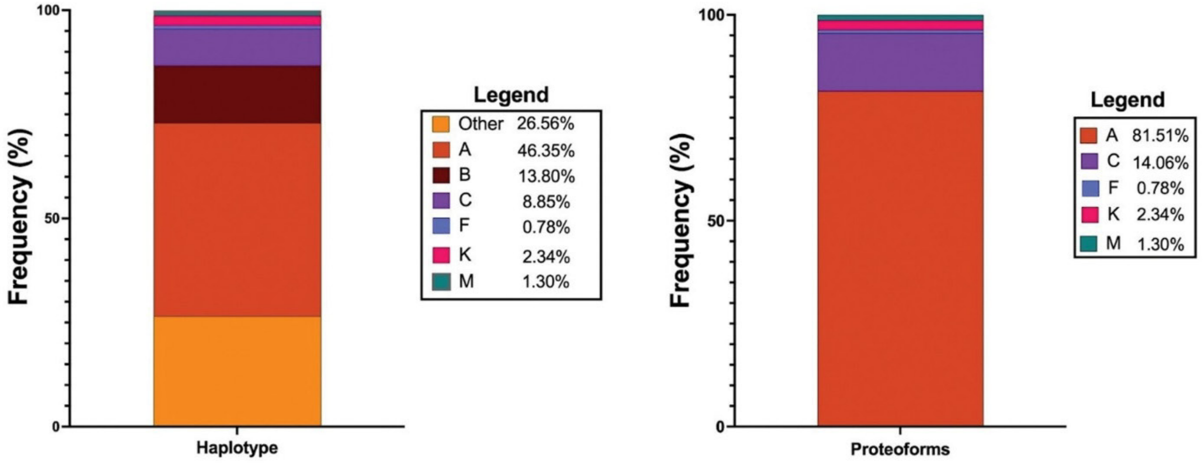

Supplemental Figure 1. Haplotype and proteoform frequencies. We identified 16 haplotypes and 5 proteoforms of the prion protein gene in white-tailed deer in the sample set (*n* = 192). All haplotypes and proteoforms identified have been previously described in the literature. Haplotypes A, B, C, F, K, and M were the most common haplotypes identified; all other haplotypes detected at lower frequencies were categorized as “other,” including haplotypes D, E, G, I, J, P, T, Ovid-27, Ovid-32, and Heaton. Only 5 proteoforms were observed, including A, C, F, K, and M. Both haplotype frequencies and proteoform frequencies were calculated by dividing the number of observed haplotypes (or proteoforms) by the total number of alleles in the sample set.

See also **Supplemental Excel rtQuIC data for all results.**
